# Spontaneous rupture of an infected renal cyst and external drainage through a lumbar surgical scar in a male patient with cervical spinal cord injury: a case report

**DOI:** 10.1186/1752-1947-2-154

**Published:** 2008-05-14

**Authors:** Subramanian Vaidyanathan, Peter L Hughes, Tun Oo, Bakul M Soni

**Affiliations:** 1 Regional Spinal Injuries Centre, District General Hospital, Southport, PR8 6PN, UK; 2 Department of Radiology, District General Hospital, Southport, PR8 6PN, UK

## Abstract

**Introduction:**

The spontaneous rupture of an infected renal cyst is a rare event. Spontaneous rupture with drainage to the exterior through a surgical scar has not been reported previously.

**Case presentation:**

A 49-year-old male with tetraplegia had undergone extended right pyelolithotomy in 1999. Deroofing and marsupialisation of a cyst in the upper pole of the right kidney was performed in 2003. Subsequently there was recurrence of a thick-walled cystic space-occupying lesion in the upper pole of the right kidney. Thick pus was aspirated from the renal cyst on six occasions between September 2003 and November 2004. In March 2006, ultrasound examination revealed a cyst measuring 6.2 cm in diameter in the upper pole of the right kidney. Aspiration was planned when the renal cyst reached 7.5 cm in diameter. However, 11 months later, the cyst ruptured spontaneously and drained through the previous surgical scar in the flank, while the patient was recovering from a severe chest infection in the spinal unit. Ultrasound examination showed a fistulous tract running between the renal cyst and the abdominal wall. Repeated minor trauma sustained during turning, hoisting and chest physiotherapy all may have contributed to the rupture of the infected renal cyst and drainage through a weak spot in the abdominal wall.

**Conclusion:**

In hindsight, we might have prevented rupture of the renal cyst had we considered aspiration of the renal cyst before it reached 7.5 cm in diameter, although this 7.5 cm diameter, as the threshold for percutaneous aspiration, is an arbitrary setting. This patient could have been advised to wear an abdominal corset to protect the right flank from pressure applied unintentionally during turning, hoisting or assisted coughing.

## Introduction

Spontaneous rupture of a renal cyst is a rare event that may occur into the pelvicalyceal system, the perirenal space, or the peritoneal cavity [1]. We present the case of a tetraplegic patient in whom an infected renal cyst ruptured spontaneously through a surgical scar in the ipsilateral flank. A search in PubMed revealed no other reports of rupture of the renal cyst to the exterior through a surgical scar either in ambulatory individuals or in persons with a spinal cord injury.

## Case presentation

**Figure 1:**
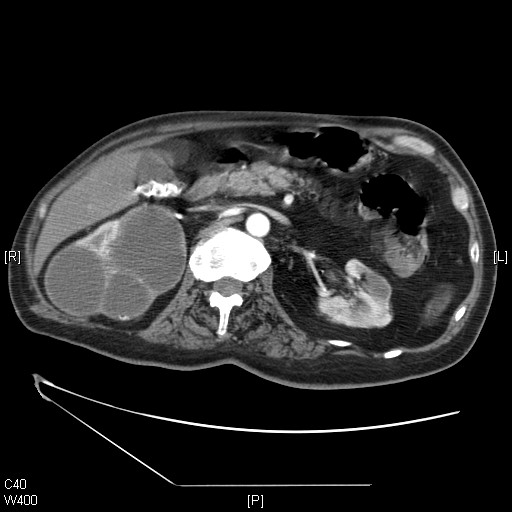
**Axial CT scan of the abdomen, performed on 7 July 2003**. Several cysts are shown in the right kidney, the largest measuring 6 cm in diameter. The outline of the right kidney was intact. The perirenal fat was seen without any discontinuity. The gall bladder contained several stones.

A 49-year old male sustained C-6 complete tetraplegia in 1975. He underwent extended right pyelolithotomy in 1999. In 2003, this patient noticed a lump on the right side of the abdomen. A computed tomography (CT) scan of the abdomen revealed several large cysts in the right kidney (Figure 1). Open surgical deroofing and marsupialisation of a large cyst in the upper pole of the right kidney were carried out in August 2003. Subsequently, there was recurrence of a thick-walled cystic space-occupying lesion in the upper pole of the right kidney containing reflective fluid, which was consistent with an infected cyst. Thick pus was aspirated from the infected renal cyst on six occasions since September 2003; the last aspiration was performed in November 2004. Microbiology of the aspirate revealed the growth of coliforms. In March 2006, an ultrasound scan revealed a cyst measuring 6.2 cm in diameter in the upper pole of the right kidney. The wall of the cyst was mildly, uniformly thickened with no irregularity. The cyst contained clear fluid. There was a further adjacent 3 cm simple cyst at the upper pole of the right kidney with associated calculi. Gallstones were noted in the gallbladder. The left kidney contained small cysts. As there was no clinical or sonographic evidence of infection in the right renal cysts, the patient was kept under observation.

**Figure 2:**
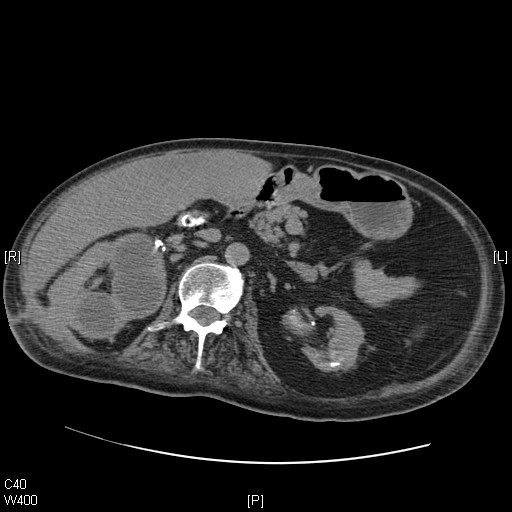
**Axial CT scan of the abdomen, performed on 5 March 2007**. Cysts in upper pole of right kidney are shown, the largest measuring 7 cm. In contrast to Figure 1, there was a marked loss of fat plane between the mid-pole of the right kidney and the postero-lateral abdominal wall.

**Figure 3:**
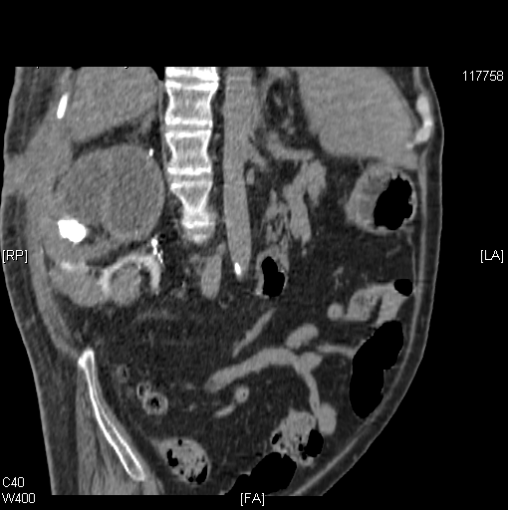
**Sagittal reconstruction of the CT scan of the abdomen, performed on 5 March 2007**. Communication between the cyst in the right kidney and the exterior through a defect in the anterior abdominal wall musculature is revealed.

In November 2006, the patient developed chest infection and required mechanical ventilation. During this admission, medical care was focused on the patient's chest condition, and understandably the renal cyst was not given priority in investigation or treatment. In February 2007, the patient had been weaned off the ventilator but he was still requiring intense chest physiotherapy. While turning the patient, nurses noticed a swelling in the middle of the right flank scar. After two days, the swelling burst and purulent material drained on to the skin. Microbiology of the purulent discharge revealed the growth of *Providencia stuartii*. This patient did not develop a fever, shivering or any other indicative feature of infection. Ultrasound examination showed a fistulous tract running between the renal cyst and the abdominal wall. A CT scan of the abdomen was performed to look for any collection of pus in the perinephric space. The CT scan revealed loss of the fat plane between the mid-pole of the right kidney and the postero-lateral abdominal wall (Figure 2). This was contiguous with a 4 × 2 cm area of soft tissue thickening and fluid collection in the subcutaneous fat. There was a small pocket of gas. There was extruded calcific material from the right kidney antero-lateral to the psoas muscle. On sagittal reconstruction of the CT scan, a communication between the cyst in right kidney and exterior through a defect in abdominal muscles could be seen distinctly (Figure 3). The amount of purulent discharge draining through the flank wound decreased gradually over a period of 10 weeks. The patient remained afebrile. This patient was scheduled for aspiration of the remaining cysts in the right kidney once the infective process subsided.

## Discussion

A simple renal cyst is the most common abnormality observed during routine ultrasound examination of the kidneys in asymptomatic spinal cord injury patients, and does not usually warrant any intervention [2]. A renal cyst may be infected, albeit rarely. An infected renal cyst can be distinguished from a simple renal cyst by magnetic resonance imaging, as an infected renal cyst is less intense than a simple renal cyst on T2 weighted imaging [3]. An infected renal cyst requires drainage without delay [4]; otherwise, the infected cyst may rupture due to increased intracystic pressure as well as weakening of the thin tissue walls separating the cyst from a closely adjacent collecting system or perinephric space [5]. Very rarely, rupture of an infected renal cyst may result in fatal consequences [6]. Spontaneous rupture of an infected renal cyst can pose diagnostic difficulties [7]. A patient with spontaneous rupture of infected renal cyst may present to the emergency department, with distension of the right flank occurring suddenly [8].

In the case of our patient, when the renal cyst ruptured, the surgical scar in the ipsilateral flank proved to be the path of least resistance. Drainage of infected material externally through the previous surgical scar helped to prevent systemic infection. If such a weak spot had not existed, the infected cyst might have ruptured into the retroperitoneum resulting in inflammation of the perinephric space and collection of pus, which would have required percutaneous or open surgical drainage.

Unlike ambulatory individuals, tetraplegic patients are at greater risk of sustaining blunt trauma to the abdomen during their routine activities of daily living. Paulson et al [9] reported rupture of the spleen in a tetraplegic patient, who slid sideways, catching his flank between the wheelchair arm and a slightly reclined wheelchair back. In our patient, there was no history of acute trauma to the abdomen prior to the rupture of the renal cyst. However, repeated minor trauma, sustained during routine activities of daily living, could have played a significant role in the rupture of the renal cyst in this tetraplegic patient. For example, it has been documented that men with spinal cord injury can sustain blunt trauma to the scrotum during transfers to a toilet seat or a car seat [10]. In the reported cases, the scrotum was compressed by the weight of the body during transfers or the scrotum was trapped between the thighs. Analogous to the blunt trauma to the scrotum incurred during transfers, it is conceivable that, in our patient, the renal cyst situated under the surgical scar was subjected to minor trauma during turning in bed or while hoisting. Further, the right flank might have been subjected to pressure against the side plate of the wheel chair, as the tetraplegic patient was sat up on the chair. The cyst in the right kidney would have been exposed to increased pressures during vigorous chest physiotherapy (for example, assisted coughing), which this patient required because of a severe chest infection. The cumulative effect of repeated minor trauma possibly led to rupture of the infected renal cyst in this patient through a weak spot, that is, a previous surgical scar in the flank.

From this case, we have learnt the following lessons.

• We should have recognised that the renal cyst was vulnerable to rupture, and should have carried out aspiration of the renal cyst before it reached 7.5 cm in diameter, although a size of 7.5 cm in diameter as the threshold for percutaneous aspiration is an arbitrary setting. This would have prevented rupture of the renal cyst.

• We should have advised this patient to wear an abdominal corset, in order to protect the renal cyst and the surgical scar in the flank from pressures applied unintentionally to the lumbar region during hoisting or assisted coughing. We already use abdominal corsets in thin patients, in whom a Medtronic pump has been implanted in the abdominal wall for continuous intrathecal delivery of baclofen.

## Conclusion

We have reported the case of a tetraplegic patient in whom an infected cyst in the upper pole of the right kidney ruptured spontaneously and drained its contents to the exterior through a surgical scar in the ipsilateral flank. Spontaneous rupture of a renal cyst is a very rare event and external drainage through a previous surgical scar has not been reported previously in persons with spinal cord injury. This tetraplegic patient possibly sustained repeated minor trauma to his flank during turning, hoisting and chest physiotherapy, which contributed to the rupture of the renal cyst. In hindsight, we should have anticipated that the renal cyst was at risk of rupture and should have observed precautionary measures, such as aspiration of the cyst even before it reached 7.5 cm in diameter. Further, this patient should have been prescribed an abdominal corset to protect the renal cyst and the surgical scar from pressures applied unintentionally to the lumbar region during turning, hoisting and chest physiotherapy.

## Competing interests

The authors declare that they have no competing interests.

## Authors' contributions

SV developed the concept for this case report and wrote the draft. PLH provided the medical images. TO provided clinical care. All authors contributed to the final manuscript.

## Consent

Written informed consent was obtained from the patient for publication of this case report and accompanying images. A copy of the written consent is available for review by the Editor-in-Chief of this journal.

## References

[B1] Nussbaum A Hunter TB Stables DP Spontaneous cyst rupture on renal CT AJR Am J Roentgenol 1984 142 751 752 6608236 10.2214/ajr.142.4.751

[B2] Vaidyanathan S Hughes PL Soni BM A comparative study of ultrasound examination of urinary tract performed on spinal cord injury patients with no urinary symptoms and spinal cord injury patients with symptoms related to urinary tract: do findings of ultrasound examination lead to changes in clinical management? Scientific World Journal 2006 6 2450 2459 17619717 10.1100/tsw.2006.382 PMC5917309

[B3] Takashima M Miyazaki K Asari T Fujita Y Ikeda D Yoshida M A case of infected renal cyst: the usefulness of magnetic resonance imaging for preoperative diagnosis Hinyokika Kiyo 1993 39 837 839 8213379

[B4] Koh E Kondoh N Kiyohara H A case of infected solitary renal cyst treated with percutaneous puncture and drainage Hinyokika Kiyo 1991 37 381 384 1891996

[B5] Papanicolaou N Pfister RC Yoder IC Spontaneous and traumatic rupture of renal cysts: diagnosis and outcome Radiology 1986 160 99 103 3715054 10.1148/radiology.160.1.3715054

[B6] Finlay DB Lowe JS Kaur K Perforation of a suppurative solitary renal cyst Br J Surg 1981 68 585 586 10.1002/bjs.1800680819 7272680

[B7] Yoshinaga A Hayashi T Ishii N Yoshida S Ohno R Terao T Watanabe T Yamada T A case of spontaneous rupture of infectious renal cyst with difficulty in diagnosis Hinyokika Kiyo 2005 51 257 259 15912785

[B8] Tokuchi H Yamamoto M Kamoto T Spontaneous rupture of infected renal cyst presenting sudden onset of right flank distension: a case report Hinyokika Kiyo 2004 50 323 326 15237485

[B9] Paulson SM Hatvani C Long C Splenic rupture and splenectomy due to fall from wheelchair Arch Phys Med Rehabil 1983 64 180 6838347

[B10] Vaidyanathan S Soni BM Singh G Subramaniam R Bingley J Sett P Parsons KF Blunt trauma to scrotum in men with spinal cord injury after they had completed rehabilitation in a spinal unit Spinal Cord 2001 39 442 448 10.1038/sj.sc.3101175 11512075

